# The Male-Produced Aggregation-Sex Pheromone of the Cerambycid Beetle *Plagionotus detritus* ssp. *detritus*

**DOI:** 10.1007/s10886-018-1031-4

**Published:** 2018-11-09

**Authors:** Mikael A. Molander, Jimmy Helgesson, Inis B. Winde, Jocelyn G. Millar, Mattias C. Larsson

**Affiliations:** 1 Unit of Chemical Ecology, Department of Plant Protection Biology, Swedish University of Agricultural Sciences, Box 102, Sundsvägen 14, 230 53 Alnarp, Sweden; 2 Nordens Ark Foundation, Åby Säteri, 456 93 Hunnebostrand, Sweden; 3 Departments of Entomology and Chemistry, University of California, Riverside, CA 92521 USA

**Keywords:** Longhorn beetle, Red list, (*R*)-3-hydroxy-2-hexanone, (*S*)-2-hydroxy-3-octanone, Monitoring, Conservation

## Abstract

The number of longhorn beetles with confirmed aggregation-sex pheromones has increased rapidly in recent years. However, the species that have been studied most intensively are pests, whereas much less is known about the pheromones of longhorn beetles that are rare or threatened. We studied the cerambycid beetle *Plagionotus detritus* ssp. *detritus* with the goal of confirming the presence and composition of an aggregation-sex pheromone. This species has suffered widespread population decline due to habitat loss in Western Europe, and it is now considered threatened and near extinction in several countries. Beetles from a captive breeding program in Sweden were used for headspace sampling. Gas chromatography-mass spectrometry revealed that collections from males contained large quantities of two compounds, identified as (*R*)-3-hydroxy-2-hexanone (major component) and (*S*)-2-hydroxy-3-octanone (minor component), in addition to smaller quantities of 2,3-hexanedione and 2,3-octanedione. None of the compounds was present in collections from females. When tested singly in a field bioassay, racemic 3-hydroxy-2-hexanone and 2-hydroxy-3-octanone were not attractive to *P. detritus*, whereas a 5:1 blend elicited significant attraction. Both compounds are known as components of the pheromones of conspecific beetles, but, to our knowledge, this is the first cerambycid shown to use two compounds with different chain lengths, in which the positions of the hydroxyl and carbonyl functions are interchanged between the two. The pheromone has potential as an efficient tool to detect and monitor populations of *P. detritus*, and may also be useful in more complex studies on the ecology and conservation requirements of this species.

## Introduction

Longhorn beetles (Cerambycidae) are a diverse, species-rich group of primarily saproxylic insects, which use long-distance sex and aggregation-sex pheromones extensively for locating mates (Allison et al. 2004; Hanks and Millar 2016; Millar and Hanks 2017). Some longhorn beetles can be severe pests and can be particularly devastating as invasive species when introduced into new areas of the world (Haack 2017; Hanks and Millar 2016). Pest species typically attack healthy, living trees or trees under stress, with major negative economic consequences to natural forests, ornamental trees, and orchards (Allison et al. 2004; Haack 2017). Pheromones can serve as valuable tools to increase the probability of early detection of invasive pest species so that rapid responses can be initiated to contain and hopefully eradicate the invaders (Hanks and Millar 2016). Consequently, in recent years, the chemical ecology of pest longhorn beetle species has been increasingly studied, resulting in the identification of attractant pheromones used by a number of these species (Millar and Hanks 2017; Xu et al. 2017).

However, the vast majority of the ~35,000 described species of longhorn beetles (Švácha and Lawrence 2014) are of little immediate economic importance because their larvae develop in woody tissues that are already dead and decaying at the time of colonization by the beetles. Nevertheless, these species are of great ecological and conservation interest as an integral part of forest ecosystems, where they function as decomposers (Edmonds and Eglitis 1989; Evans et al. 2007), serve as sources of food for other taxa (e.g. Hogstad and Stenberg 1997), and create microhabitats for additional biodiversity (Allison et al. 2004; Buse et al. 2008). Due largely to modern forestry practices, in which the critical woody substrates and habitats of these species are removed or destroyed, many longhorn beetles have suffered population reductions and shrinking distributions, which have been well documented for some species (Jeppsson et al. 2010; Klausnitzer et al. 2003; Michalcewicz and Ciach 2015). In fact, a substantial fraction of the total number of longhorn beetle species in Europe are on the European Red List of saproxylic beetles (Cálix et al. 2018).

To date, practical applications of insect pheromones have focused primarily on pest species, but attractant pheromones could also be useful tools for detection and monitoring of threatened species, and in ecological studies focusing on these species (Larsson 2016; Larsson and Molander 2016). Much of the research on threatened saproxylic insect species which has exploited pheromone-based sampling methods has targeted two beetle species which inhabit hollow tree cavities, the hermit beetle (*Osmoderma eremita*) and the large red click beetle (*Elater ferrugineus*) (Andersson et al. 2014; Kadej et al. 2015; Larsson and Svensson 2009; Svensson et al. 2011). Pheromones have only been identified from a few rare or threatened species of longhorn beetles, such as the banded alder borer *Rosalia funebris* (Ray et al. 2009) and the valley elderberry beetle *Desmocerus californicus dimorphus* (Ray et al. 2014) in North America. More recently, the pheromone of the European alpine longicorn *Rosalia alpina* was identified (Žunič Kosi et al. 2017). This species is included in the European Union’s directive on the conservation of habitats, flora and fauna (Council of Europe 1992). Because longhorn beetles display a large diversity in terms of species-specific habitat requirements such as tree species, climatic preferences, and diameter and state of decomposition of the wood (Haack 2017), it would be useful to identify the pheromones of additional threatened species which can serve as models for different ecological niches (Larsson et al. 2009; Larsson 2016).

The cerambycid genus *Plagionotus* Mulsant 1842, within the subfamily Cerambycinae, is represented by three species in north and central Europe (Löbl and Smetana 2010; Özdikmen and Turgut 2009). One of these, *Plagionotus detritus* ssp. *detritus* (Linnaeus 1758), is patchily distributed, and populations have declined in parts of its range due to loss of appropriate habitat (Ehnström 2005; Esser 2017; Lindhe et al. 2010; Niehuis 2001). The species is now listed on national and regional Red Lists in several European countries (e.g. ArtDatabanken 2015; Binot et al. 1998; Jäch 1994; Jørum et al. 1997; Monnerat et al. 2016). Besides the nominate subspecies that occurs in Europe, three other subspecies are known from Asia (Danilevsky 2018). Larvae of *P. detritus* develop in sun-exposed, recently dead oak wood of large dimensions, such as on the main trunk or thick branches (Bense 1995; Ehnström 2005; Klausnitzer et al. 2016; Lindhe et al. 2010), a substrate which is rare in modern production forests. In Sweden, the species is near regional extinction and has displayed one of the most dramatic reductions in distribution that have been recorded for any insect species (Ehnström 2005), being assigned to the threat category “endangered” (EN) on the national Red List (ArtDatabanken 2015). The Swedish Environmental Protection Agency has developed a national action plan for the long-term preservation and population recovery of the species (Ehnström 2005). Due to its scarcity and dependence on a transient substrate, it is difficult to detect and monitor *P. detritus* with conventional survey methods. Pheromone-baited traps for *P. detritus* could be used as tools to conduct standardized, quantitative field studies. The resulting data on its distribution and population densities would be of use in enhancing conservation efforts at its remaining sites in Sweden, as well as elsewhere in Western Europe.

The aim of this study was to collect headspace volatiles from *Plagionotus detritus*, to identify candidate pheromone components and to evaluate these for attractiveness in field bioassays. Furthermore, we highlight the significant potential for applying this aggregation-sex pheromone as a cost-efficient, systematic tool in non-lethal pheromone-based field studies of *P. detritus* to survey remaining populations, to monitor ongoing reintroductions of the species, and to increase our general knowledge of the species’ ecology.

## Methods and Materials

### Source of Beetles

As part of the Swedish national action plan for *P. detritus*, a captive population of the species has been established, which could be used to reintroduce the species at sites in southern Sweden where it is presumed to be extinct (Ehnström 2005). The captive population was started with individuals from the Stockholm region, using colonized wood material which was collected and transported to Nordens Ark near Gothenburg (a zoo specializing in captive breeding of endangered animal species). At Nordens Ark, newly emerged individuals were sexed based on the length of the antennae in relation to the total body length as males have markedly longer antennae than females (Bense 1995; Ehnström and Holmer 2007). Members of each sex were placed in separate containers. Before collecting volatiles, males and females were kept separately in a room with natural daylight and a temperature of 20–25 °C. They were provided with pieces of fresh oak branches and a piece of paper saturated with nectar solution prepared from a teaspoon of nectar powder for birds (Versele-Laga, Deinze, Belgium) dissolved in 100 ml tap water, which the beetles would feed on sporadically. After collecting headspace volatiles, the beetles were released back into the captive breeding population.

### Collection of Beetle Volatiles

For collection of headspace volatiles, beetles were transferred to another room with the same light and temperature conditions as above. Between four and six beetles of each gender were placed in two separate 5 L oblong glass jars (inner diameter 12 cm) fitted with tightly sealed metal lids, with holes for incoming and outgoing air. Air was pumped out of the containers through holes fitted with adsorption columns consisting of Teflon® (TFE) tubing (inner diameter 3 mm, length 50 mm) containing 25 mg of the adsorbent polymer Porapak™ Q (mesh size 50–80; Supelco/Sigma-Aldrich, Munich, Germany). Polypropylene wool, secured by short pieces of smaller TFE tubing (inner diameter 1.5 mm, length 2 mm), was inserted into the main column on both sides of the adsorbent material to hold it in place. Identical columns were also attached to the holes for incoming air in order to reduce the quantity of volatile compounds present in the air entering the system. Polyvinyl chloride (PVC) tubing (diameter 4 mm) connected the columns from the two chambers to a single air pump (model PM 10879 NMP 03; KNF Neuberger, Freiburg, Germany), which pulled ambient air through the aeration chambers. A flow meter (Kytölä Instruments, Muurame, Finland) and plastic valves on the PVC tubing were used to achieve a constant flow rate of about 0.2 l/min through each column. In total, eight aerations were made, each lasting for about 5 hr, between approximately 10:00 and 15:00 hr, when the beetles are typically active under natural conditions (Swedish Species Observation System 2015). The glass jars were reused after being rinsed with ethanol followed by acetone, and drying overnight. When starting a new aeration, male and female beetles were placed in the opposite jar compared to the previous session to avoid systematic errors.

The loaded collectors were extracted with 300 μl of hexane. A slight positive air pressure, created with a plastic syringe, was used to push the solvent through the collector and into glass vials (volume: 1.5 ml) with butyl-polytetrafluoroethylene screw caps (articles 11090210 and 08151653, Skandinaviska Genetec AB, Stockholm, Sweden). Samples were stored in a freezer at −18 °C until used in analyses. The adsorption columns were cleaned by treating them with 3 × 300 μl of hexane and 3 × 300 μl of acetone before reuse.

### Identification of Pheromone Components

Aeration samples were analyzed by coupled gas chromatography-mass spectrometry (GC-MS) using a 6890 N GC interfaced to a 5975 mass selective detector (Agilent Technologies, Santa Clara, CA, USA) at SLU Alnarp. The GC was equipped with an HP-5MS column (60 m × 0.25 inner diam., 0.25 μm film thickness, Agilent Technologies). Manual injections of 2 μl of each aeration sample were made in splitless mode (injector temperature 225 °C, split vent opened after 0.5 min) using helium as the carrier gas (constant flow rate of 1.8 ml/min) and a transfer line temperature of 150 °C. The inlet pressure was 172 kPa and the GC oven temperature was programmed at 30 °C for 3 min, and then at 8 °C min^−1^ up to 260 °C and held for 10 min. The mass spectrometer was set to begin recording after a 6.5 min solvent delay. Mass spectra were taken in electron impact ionization (EI) mode at 70 eV, with a scanning range of 29.0–400.0 *m/z*. Sex-specific peaks were recognized by visually comparing overlaid chromatograms from males and females. The sex-specific peaks were then tentatively identified by matching their mass spectra to reference spectra in the NIST and Wiley commercial mass spectral databases or to those of previously known cerambycid pheromones. Identities were then confirmed by matching mass spectra and retention times to those of synthetic standards.

Extracts of volatiles were also analyzed at UC Riverside by GC-MS using an Agilent 7820A GC (Agilent, Santa Clara, CA, USA) interfaced with a 5977E MS. The GC was equipped with a DB-5 column (30 m × 0.25 mm diameter, 0.25 μm film thickness; J&W Scientific, Folsom CA, USA). Injections of 1 μl were made in splitless mode with an autosampler (injector temperature 250 °C, transfer line 280 °C, split vent opened after 0.5 min), with helium carrier gas (inlet pressure 89.6 kPa, linear velocity 34 cm/s). The oven was programmed from 40 °C for 1 min, and then at 10 °C min^−1^ to 280 °C. Mass spectra were taken in EI mode at 70 eV, scanning a mass range from 40.0–450.0 *m/z*, with a 3 min solvent delay.

To determine the absolute configurations of the insect-produced compounds, aliquots of the extracts were analyzed at UC Riverside on a chiral stationary phase Cyclodex B GC column (30 m × 0.25 mm i.d., 0.25 μm film thickness, J&W Scientific), with an injector temperature of 150 °C to minimize isomerization of thermally labile hydroxyketones. Injections were made in split mode (split ratio ~20:1) at 172 kPa inlet pressure, and the oven was programmed at 50 °C for 1 min, then at 3 °C/min to 220 °C, and held for 20 min. The GC was equipped with a flame ionization detector (detector temperature 250 °C), and raw data were processed with Peak Simple software (SRI Instruments, Torrance, CA, USA). Authentic standards were analyzed under the same conditions, and the identification of the insect-produced enantiomer of each hydroxyketone was confirmed by coinjection of the insect extract with the racemic standards, determining which of the two peaks in each racemate was enhanced.

### Sources of Chemicals

Although the beetles were shown to produce pure (*R*)-3-hydroxy-2-hexanone and (*S*)-2-hydroxy-3-octanone (see Results), we used racemic mixtures of both compounds in bioassays because enantiomerically pure compounds could not be synthesized in the quantities needed in time for the field bioassay. Racemic 3-hydroxy-2-hexanone (CAS number 54123–75-0) was obtained from Bedoukian Research (Danbury, CT, USA) and racemic 2-hydroxy-3-octanone was synthesized from methyl lactate (TCI America, Portland, OR, USA) using methods previously described (Hall et al. 2006). Synthetic 2,3-hexanedione was purchased from Aldrich Chemical Co. (Milwaukee, WI, USA), and a reference sample of 2,3-octanedione was generated by oxidation of a few mg of (*S*)-2-hydroxy-3-octanone, available from previous work, with pyridinium dichromate in methylene chloride.

### Field Bioassay

Treatments consisted of racemic 3-hydroxy-2-hexanone (100 mg/ml in isopropanol) and racemic 2-hydroxy-3-octanone (100 mg/ml) as single components and as a 5:1 blend (100 mg/ml racemic 3-hydroxy-2-hexanone and 20 mg/ml racemic 2-hydroxy-3-octanone), mimicking the average ratio found in aeration samples (see Results). The control consisted of isopropanol alone. A 0.5 ml aliquot of each solution was loaded into a polyethylene Grippie® zip-lock bag (5.5 × 6.5 cm × 40 μm, Grippie Light Nr-02, b.n.t. Scandinavia AB, Arlöv, Sweden) at the field site. Each bag was then sealed and attached to the south-facing, central part of the trap using metal wire. We excluded 2,3-hexanedione and 2,3-octanedione from our bioassay because these compounds are likely to be artefacts from degradation of the corresponding ketols in the aeration samples (Millar and Hanks 2017).

**Fig. 1:**
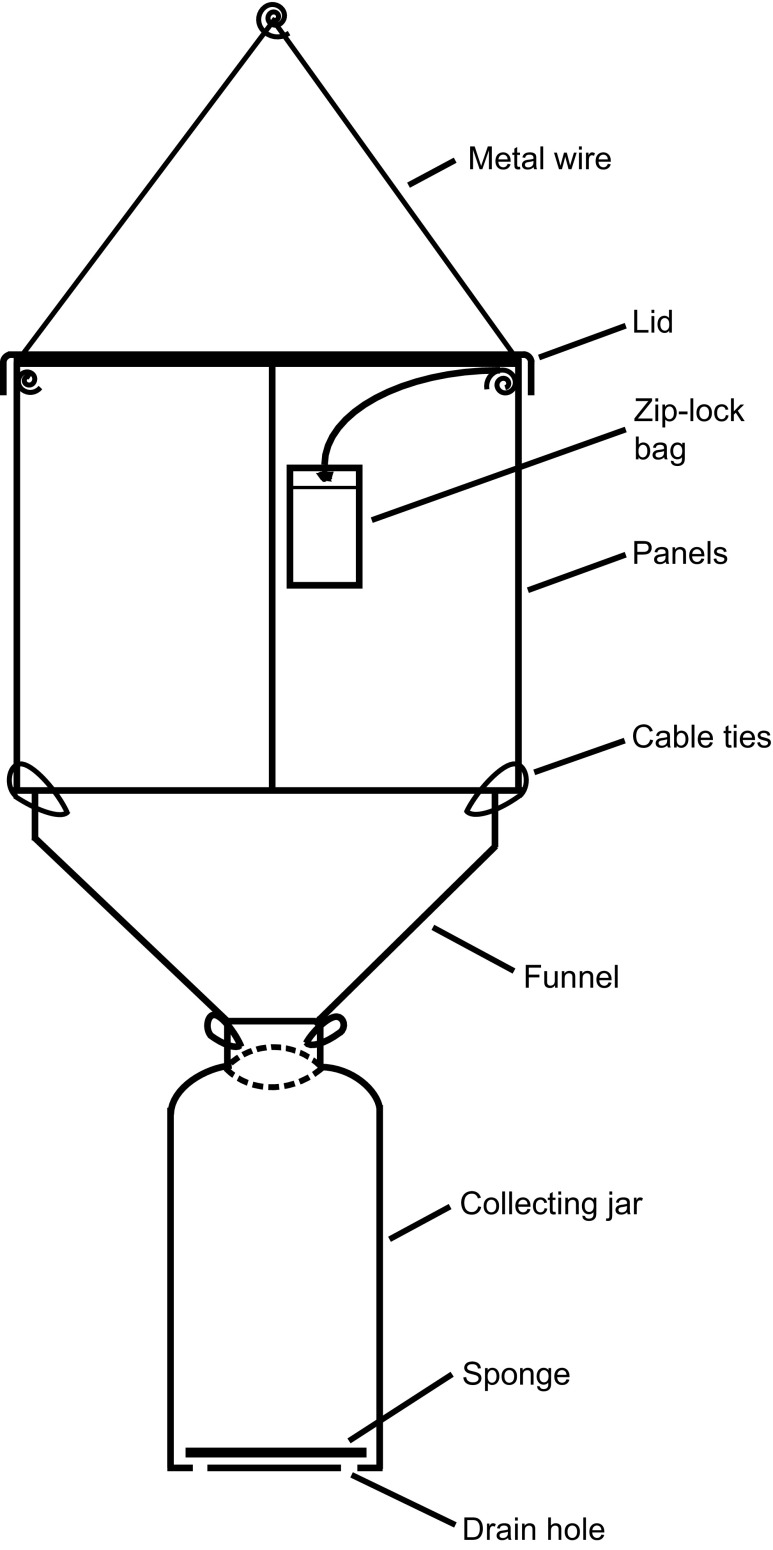
Schematic drawing of the cross-vane flight-intercept trap used in the field bioassay (seen from the side, hanging vertically)

Custom-made cross-vane panel flight-intercept traps were used in the bioassay (Fig. 1). The rectangular black panels were 20 cm tall and 25 cm wide (Nordic Plastics Group AB, Trelleborg, Sweden), and the black plastic funnel placed below the crossed panels had a top diameter of 20 cm and slope of about 45 degrees (Hall Miba, Alvesta, Sweden). The beetles collided with the panels and slid down the funnel into a 1 L white plastic collecting jar (Corning Life Science, Stockholm, Sweden). A circular roof (brown color, diameter: 28 cm; Soparco, Chaingy, France) served as protection from rain. Wire and cable ties were used to assemble the different parts. To increase trap efficiency, the panels, the inside of the funnel, and the inside of the trap jar were treated with fluon (polytetrafluoroethylene dispersion, 60 wt% in H_2_O, further diluted 1:1 with water; Sigma-Aldrich, Saint Louis, MO, USA) (Graham and Poland 2012). A thin piece of sponge was placed at the bottom of each collection jar and almost saturated with water each time the trap was emptied to provide trapped beetles with moisture and decrease the risk of desiccation. Four small holes were drilled in the bottom of the jar to let excess water escape in case of rain.

The field bioassay was performed at Stora Skuggan in Djurgården Royal National City Park, Stockholm (center coordinates: DD 59.3632 / 18.0800). The test site was a south-facing hill slope with a mixture of mostly deciduous trees, including a number of large, old oaks. Parts of the site are used as a “tree cemetery” where the park management retains dead wood (particularly oak) from other parts of the Royal City Park to benefit saproxylic invertebrates (Ehnström 2005). *Plagionotus detritus* is observed regularly, and often in numbers, at this site (Ehnström 2005; Swedish Species Observation System 2015). Three replicates (separated by about 100 m), each with four traps (one for each lure treatment), were deployed in the afternoon on June 29, 2015. The traps were spaced ~20 m apart, hanging from branches in sunny glades or forest edges at a height of ~1.8 m. The positions of traps within each replicate were randomized when the traps were initially deployed. The traps were then emptied in the early evening for 5 consecutive days from June 30–July 4, generating a total of five observations per trap and 15 observations per treatment. Trapped beetles were counted and sexed, and then released ~5 m from the position where they had been captured. Beetles were released rather than being killed because of their endangered status. At each count, the traps were moved one position clockwise to minimize position effects.

### Statistical Analysis

Data from the bioassay were not normally distributed and exhibited heteroscedasticity. Thus, the nonparametric *Kruskal-Wallis H test* was used to compare numbers of beetles caught per treatment (converted to mean ranks). Pairwise multiple comparisons post hoc tests were carried out using *Dunn’s test* (1964), with a Bonferroni correction to adjust the significance values for multiple comparisons. All calculations were performed in RStudio Desktop, version 1.1.453 for Windows (RStudio 2018).

## Results

### Pheromone Identification

**Fig. 2:**
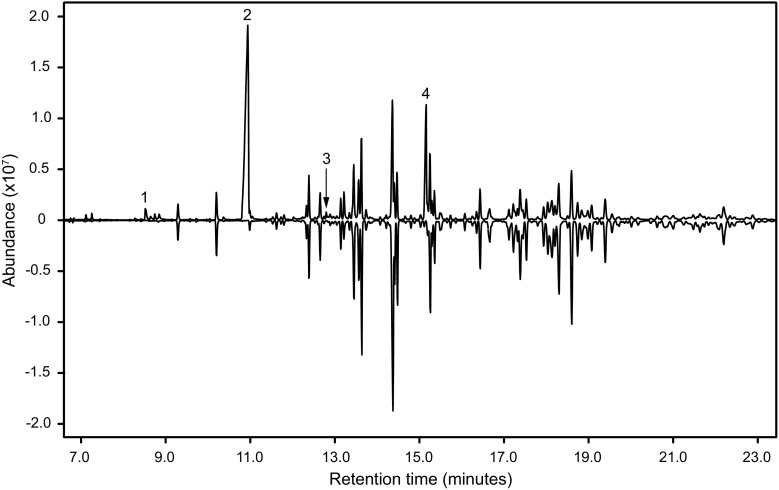
Representative total ion chromatograms (HP-5 column) of headspace volatiles collected from adult male (top trace) and female *P. detritus* (bottom, inverted trace). Four compounds, unique to samples from males, are denoted by numbers; 2,3-hexanedione (1), (*R*)-3-hydroxy-2-hexanone (2), 2,3-octanedione (3) and (*S*)-2-hydroxy-3-octanone (4). Remaining compounds present in samples from both males and females are system contaminants

Analysis of the headspace extracts from males and females by GC-MS showed that six out of the eight extracts from males contained comparatively large quantities of between two and four insect-produced compounds (Fig. 2). The compounds were readily identified as 2,3-hexanedione, 3-hydroxy-2-hexanone, 2,3-octanedione, and 2-hydroxy-3-octanone by matches with database spectra (2,3-hexanedione and 2,3-octanedione), and by matching their retention times and mass spectra to standards of the previously known cerambycid pheromone compounds for 3-hydroxy-2-hexanone and 2-hydroxy-3-octanone.

Analysis with the chiral column determined that the insects produced (*R*)-3-hydroxy-2-hexanone and (*S*)-2-hydroxy-3-octanone. Six samples from males contained (*R*)-3-hydroxy-2-hexanone and five samples had lesser quantities of 2,3-hexanedione, whereas five samples contained (*S*)-2-hydroxy-3-octanone and three of the five samples also contained trace amounts of 2,3-octanedione. The quantity of (*S*)-2-hydroxy-3-octanone in the five samples that contained this compound was consistently lower (on average 22%) than that of (*R*)-3-hydroxy-2-hexanone, but the proportions fluctuated (minimum 16%, maximum 52%). The samples from females did not contain any of these compounds, nor did they contain any female-specific compounds.

### Field Bioassay

**Fig. 3:**
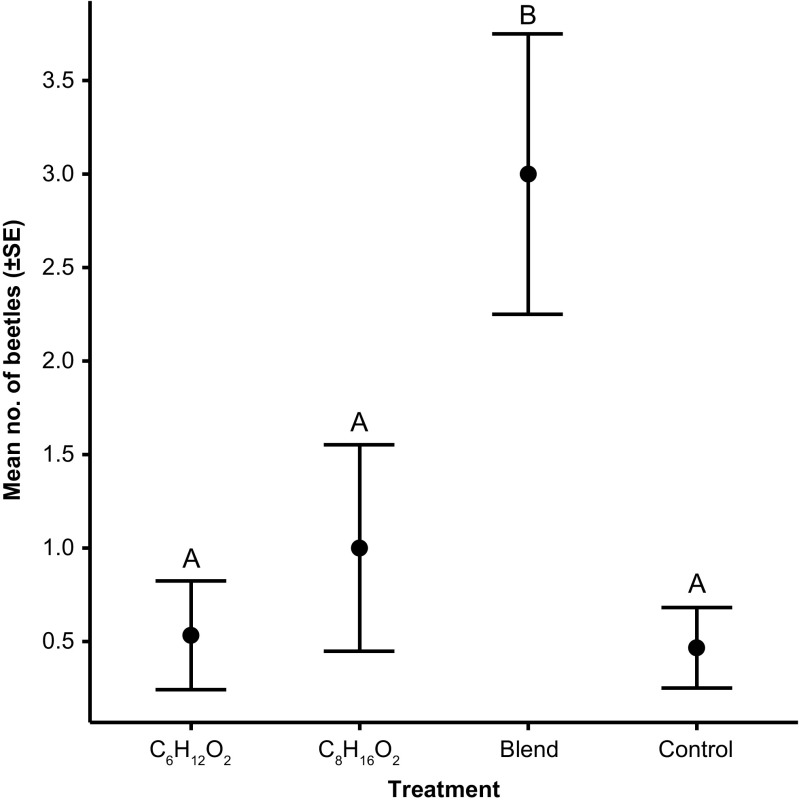
Mean (± 1 SE) numbers of *P. detritus* captured per collection date in traps with different lure compositions (*n* = 15 samples per treatment): C_6_H_12_O_2_ (racemic 3-hydroxy-2-hexanone; 50 mg/bait in 0.5 ml isopropanol); C_8_H_16_O_2_ (racemic 2-hydroxy-3-octanone; 50 mg/bait in 0.5 ml isopropanol); Blend (5:1 blend of racemic 3-hydroxy-2-hexanone at 50 mg/bait and racemic 2-hydroxy-3-octanone at 10 mg/bait in 0.5 ml isopropanol); Control (0.5 ml isopropanol). Means with different letters are significantly different (*P* < 0.05)

In total 75 *Plagionotus detritus* were captured in the bioassay. The lure treatment had an overall significant effect (*Kruskal-Wallis*: *χ*^*2*^ = 19.9, 3 *d.f.*, *P* < 0.001) (Fig. 3). Catches with the individual compounds were not statistically different from those in the control (post hoc: *Z* = −0.270, adjusted *P* = 1.0 and *Z* = 0.305, Adj. *P* = 1.0). However, the blend of the two compounds attracted significantly more beetles than the control (post hoc: *Z* = 3.62, Adj. *P* = 0.002) and the individual compounds (post hoc: *Z* = −3.89, Adj. *P* < 0.001 and *Z* = −3.32, Adj. *P* = 0.005). The total catch from the blend comprised 56% males (25 individuals) and 44% females (20 individuals).

## Discussion

The analytical results and bioassay data demonstrate that *Plagionotus detritus* uses a two-component male-produced aggregation-sex pheromone. The two compounds acted synergistically, with neither being significantly attractive as a single component. Although there was considerable variation in the relative amounts of the two compounds in the extracts of volatiles, (*S*)-2-hydroxy-3-octanone was always the minor component. Imrei et al. (2014) had briefly reported the identification and testing of these two compounds with Hungarian populations of *P. detritus*, but their detailed results apparently have not yet been published. Also, Flaherty et al. (2018) and Rassati et al. (2018) tested “*multi-lures*” containing blends of racemic 3-hydroxy-2-hexanone, racemic 3-hydroxy-2-octanone, and *syn*-2,3-hexanediols at field sites in Poland and Italy. The multi-lures were significantly more attractive to *P. detritus* compared to traps baited with ultra-high release ethanol or other blends of common pheromones and/or attractants of cerambycids and other saproxylic beetles. In this study, we found that (*R*)-3-hydroxy-2-hexanone is the main component of the pheromone blend for beetles from the Swedish population. To fully optimize the effectiveness of the synthetic blend, it would be useful to test a range of blend ratios, to determine which is most attractive. We did not conduct a blend ratios test due to the difficulties associated with setting up larger field trials in the urban environment of the Royal City Park in Stockholm City, where there are relatively few suitable test sites that are not heavily frequented by the public. It would also be useful to determine whether the insect-produced (*R*)-enantiomer of 3-hydroxy-2-hexanone and the (*S*)-enantiomer of 2-hydroxy-3-octanone are more attractive than the racemic compounds. However, the presence of the opposite enantiomer in general does not appear to antagonize attraction of longhorn beetles which use hydroxyketones as pheromone components (Millar and Hanks 2017), and in the recently published trials described above (Flaherty et al. 2018; Rassati et al. 2018), large numbers of *P. detritus* were attracted to multi-lures containing blends of racemic 3-hydroxy-2-hexanone.

To date, male-produced aggregation-sex pheromones have been conclusively identified from about 30 species within the subfamily Cerambycinae, and many additional species have been shown to be attracted to compounds such as 3-hydroxy-2-hexanone (for an overview see Millar and Hanks 2017). Typically, the pheromones consist of one to three compounds with even-numbered chain lengths of six to ten carbons and either ketone or alcohol functions in the second or third position (Allison et al. 2004; Millar and Hanks 2017). However, more recently, additional structures have been identified from other species in the subfamily, such as a diketopyrrole and various alcohols and aldehydes (e.g. Zou et al. 2016; Xu et al. 2017). The two compounds which are used by *P. detritus* are common constituents of pheromones for species in this subfamily. Multiple species use (*R*)-3-hydroxy-2-hexanone as a pheromone or as a component in a blend. For instance, males of the congeneric species *Plagionotus arcuatus* also produce this compound (Schröder 1996). Likewise, males of several species, particularly in the genus *Xylotrechus*, are known to produce (*S*)-2-hydroxy-3-octanone, and this compound is also produced by male *Plagionotus christophi* (e.g. Iwabuchi 1999; Schröder 1996). However, to our knowledge, *P. detritus* is the first cerambycid species that has been shown to use a combination of two compounds with different chain lengths, in which the positions of the hydroxyl and carbonyl functions are interchanged in the two components.

The pheromone should provide a sensitive, cost-effective, and non-lethal tool to detect and monitor *P. detritus* at sites in Sweden and elsewhere which still harbor populations, provided that traps can be emptied at relatively short intervals. There are also ongoing attempts to reintroduce the species at two sites in Sweden which the species used to occupy. Pheromone-based trapping will permit non-invasive, systematic follow-up studies on the reestablishment and potential spread of the species at these sites. Previous surveys have relied on passive window traps that kill the insects, or manual searches for adults and emergence holes (e.g. Eriksson 2007; Isaksson 2005). Both methods are highly time-consuming and difficult to standardize. For example, it is sometimes challenging to determine the exact age of exit holes, and visual searches for adults are typically restricted to days with warm, sunny weather. It is also problematic from a practical point of view to survey the species by collecting emerging adults from large-diameter logs collected in the field, particularly as the oviposition sites are often found high up on the trunk or in the crown region of old oaks (Isaksson 2005). Interestingly, both studies by Flaherty et al. (2018) and Rassati et al. (2018) found that pheromone traps baited with multi-lures (see above) and placed in the forest canopy captured significantly more *P. detritus* per trap than identical traps deployed in the forest understory. Thus, for optimal detection and monitoring of *P. detritus* with pheromone traps, it may be beneficial to deploy the traps in the canopy, rather than at ground level. Effects of trap color should also be considered as Rassati et al. (2018) found purple traps to be significantly more attractive to *P. detritus* than green traps.

Besides exploiting the pheromone to detect and monitor populations of *P. detritus*, it could also be used as a tool to address more complex questions associated with the ecology and conservation requirements of the species. For example, quantitative, high-accuracy data on local population sizes and detailed mapping of the area of occupancy provided by pheromone trapping data may be matched to existing large-scale background datasets on biotic and abiotic factors at the landscape level in geographical information systems (GIS). This approach could be used to develop robust models that can guide conservation efforts and management actions (cf. Musa et al. 2013). One example of such studies is presented by Rassati et al. (2018), who showed that *P. detritus* was significantly more abundant in semi-natural forests compared to reforested forests in Italy, using pheromone-based trapping with multi-lures. Non-lethal sampling with pheromones also greatly expands the possibility to conduct mark-recapture studies, to better understand the dispersal power and movement patterns of the beetles (Svensson et al. 2011), which will be essential as the species’ habitat is fragmented in Sweden, with widely separated habitat islands (ArtDatabanken 2015; Ehnström 2005). Similar mark-recapture studies, based on traps baited with pheromones or related attractants, have been undertaken for other longhorn beetles (Torres-Vila et al. 2015, 2017). Ecological studies on *P. detritus* should also include sites in other European countries to provide a more complete picture of the ecology, distribution, and population density of this species overall.

## References

[CR1] Allison JD, Borden JH, Seybold SJ (2004). A review of the chemical ecology of the Cerambycidae (Coleoptera). Chemoecology.

[CR2] Andersson K, Bergman KO, Andersson F, Hedenström E, Jansson N, Burman J, Winde I, Larsson MC, Milberg P (2014). High-accuracy sampling of saproxylic diversity indicators at regional scales with pheromones: the case of *Elater ferrugineus* (Coleoptera, Elateridae). Biol Conserv.

[CR3] ArtDatabanken (2015) The 2015 Swedish red list. Swedish Species Information Centre, Uppsala

[CR4] Bense U (1995). Longhorn beetles: illustrated key to the Cerambycidae of Europe.

[CR5] Binot M, Bless R, Boye P, Gruttke H, Pretscher P (1998). Rote Liste gefährdeter Tiere Deustchlands.

[CR6] Buse J, Ranius T, Assmann T (2008). An endangered longhorn beetle associated with old oaks and its possible role as an ecosystem engineer. Conserv Biol.

[CR7] Cálix M, Alexander KNA, Nieto A, Dodelin B, Soldati F, Telnov D, Vazquez-Albalate X, Aleksandrowicz O, Audisio P, Istrate P, Jansson N, Legakis A, Liberto A, Makris C, Merkl O, Mugerwa Pettersson R, Schlaghamersky J, Bologna MA, Brustel H, Buse J, Novák V, Purchart L (2018) European red list of saproxylic beetles. IUCN, Brussels

[CR8] Council of Europe (1992) Convention on the conservation of European wildlife and natural habitats. Document T-PVS(92)10, Strasbourg

[CR9] Danilevsky ML (2018) Catalogue of Palaearctic Cerambycoidea. Updated 05.07.2018. https://www.zin.ru/Animalia/Coleoptera/rus/danlists.htm. Accessed 29 July 2018

[CR10] Dunn OJ (1964). Multiple comparisons using rank sums. Technometrics.

[CR11] Edmonds RL, Eglitis A (1989). The role of the Douglas-fir beetle and wood borers in the decomposition of and nutrient release from Douglas-fir logs. Can J For Res.

[CR12] Ehnström B (2005). Åtgärdsprogram för bevarande av bredbandad ekbarkbock (*Plagionotus detritus*).

[CR13] Ehnström B, Holmer M (2007). Nationalnyckeln till Sveriges flora och fauna. Skalbaggar: Långhorningar. Coleoptera: Cerambycidae.

[CR14] Eriksson P (2007). Inventering av bredbandad ekbarkbock *Plagionotus detritus* i Uppsala och Kalmar län 2005.

[CR15] Esser J (2017). Rote Liste und Gesamtartenliste der Bockkäfer (Coleoptera: Cerambycidae) von Berlin.

[CR16] Evans HF, Moraal LG, Pajares JA, Lieutier F, Day KR, Battisti A, Grégoire J-C, Evans HF (2007). Biology, ecology and economic importance of Buprestidae and Cerambycidae. Bark and wood boring insects in living trees in Europe, a synthesis.

[CR17] Flaherty L, Gutowski JMG, Hughes C, Mayo P, Mokrzycki T, Pohl G, Silk P, Van Rooyen K, Sweeney J (2018) Pheromone-enhanced lure blends and multiple trap heights improve detection of bark and wood-boring beetles potentially moved in solid wood packaging. J Pest Sci. 10.1007/s10340-018-1019-4

[CR18] Graham EE, Poland TM (2012). Efficacy of fluon conditioning for capturing cerambycid beetles in different trap designs and persistence on panel traps over time. J Econ Entomol.

[CR19] Haack RA, Wang Q (2017). Cerambycid pests in forests and urban trees. Cerambycidae of the world: biology and pest management.

[CR20] Hall DR, Cork A, Phythian SJ, Chittamuru S, Jayarama BK, Venkatesha MG, Sreedharan K, Kumar PV, Seetharama HG, Naidu R (2006). Identification of components of male-produced pheromone of coffee white stemborer, *Xylotrechus quadripes*. J Chem Ecol.

[CR21] Hanks LM, Millar JG (2016). Sex and aggregation-sex pheromones of cerambycid beetles: basic science and practical applications. J Chem Ecol.

[CR22] Hogstad O, Stenberg I (1997). Breeding success, nestling diet and parental care in the white-backed woodpecker *Dendrocopus leucotos*. J Ornithol.

[CR23] Imrei Z, Millar JG, Csonka G, Orgovan E, Domingue M, Toth M (2014) Prey pheromone as a kairomone in a beetle complex of a Hungarian oak forest (Coleoptera: Cerambycidae and Cleridae). Abstract 115, joint meeting of the International Society for Chemical Ecology and the Chemical Signals in Vertebrates Group, Champaign, IL, USA, July 8–12, 2014

[CR24] Isaksson D (2005). Inventering av bredbandad ekbarkbock i Nationalstadsparken.

[CR25] Iwabuchi K, Hidaka T, Matsumoto Y, Honda K (1999). Sex pheromones of cerambycid beetles. Environmental entomology.

[CR26] Jäch MA (1994) Rote Liste der gefährdeten Käfer Österreich (Coleoptera). Bundesministeriums für Umwelt, Jugend und Familie, Vienna

[CR27] Jeppsson T, Lindhe A, Gärdenfors U, Forslund P (2010). The use of historical collections to estimate population trends: a case study using Swedish longhorn beetles (Coleoptera: Cerambycidae). Biol Conserv.

[CR28] Jørum P, Kristensen S, Mahler V, Martin O, Holmen M, Gønget H, Stoltze M, Pihl S (1997). Biller. Rødliste over planter og dyr i Danmark.

[CR29] Kadej M, Zając K, Ruta R, Gutowski JM, Tarnawski D, Smolis A, Olbrycht T, Malkiewicz A, Myśków E, Larsson MC, Andersson F, Hedenström E (2015). Sex pheromones as a tool to overcome the Wallacean shortfall in conservation biology: a case of *Elater ferrugineus* Linneus, 1758 (Coleoptera: Elateridae). J Insect Conserv.

[CR30] Klausnitzer B, Bense U, Neumann V (2003) *Cerambyx cerdo*. In: Petersen B, Ellwanger G, Biewald G, et al. (eds) Das europäische Schutzgebietssystem Natura 2000. Ökologie und Verbreitung von Arten der FFH-Richtlinie in Deutschland. Band 1: Pflanzen und Wirbellose. Landwirtschaftsverlag, Bonn-Bad Godesberg, pp 362–369

[CR31] Klausnitzer B, Klausnitzer U, Wachmann E, Hromádko Z (2016). Die Bockkäfer Mitteleuropas.

[CR32] Larsson MC (2016). Pheromones and other semiochemicals for monitoring rare and endangered species. J Chem Ecol.

[CR33] Larsson MC, Molander MA (2016). Standardized pheromone-based toolboxes of saproxylic indicator species guiding European conservation efforts?. Bulletin de la Société Royale Belge d’Entomologie van de Koninklijke Belgische Vereiniging voor Entomologie.

[CR34] Larsson MC, Svensson GP (2009). Pheromone monitoring of rare and threatened insects: exploiting a pheromone-kairomone system to estimate prey and predator abundance. Conserv Biol.

[CR35] Larsson MC, Svensson GP, Ryrholm N, Samways MJ, McGeoch MA, New TR (2009). Monitoring rare and threatened insects with pheromone attractants. Insect conservation, a handbook of approaches and methods.

[CR36] Lindhe A, Jeppsson T, Ehnström B (2010) Longhorn beetles in Sweden – changes in distribution and abundance over the last two hundred years. Entomologisk Tidskrift 131:241–510

[CR37] Löbl I, Smetana A (2010). Catalogue of Palearctic Coleoptera, volume 6, Chrysomeloidea.

[CR38] Michalcewicz J, Ciach M (2015). Current distribution of the Rosalia longicorn *Rosalia alpina* (Linnaeus 1758) (Coleoptera: Cerambycidae) in Poland. Polish J Entomol.

[CR39] Millar JG, Hanks LM, Wang Q (2017). Chemical ecology of cerambycids. Cerambycidae of the world: biology and pest management.

[CR40] Monnerat C, Barbalat S, Lachat T, Gonseth Y (2016) Liste rouge des Coléoptères Buprestidés, Cérambycidés, Cétoniidés et Lucanidés. Espèces menaces en Suisse. Office Fédéral de l’Environment, Bern

[CR41] Musa N, Andersson K, Burman J, Andersson F, Hedenström E, Jansson N, Paltto H, Westerberg L, Winde I, Larsson MC, Bergman K-O, Milberg P (2013). Using sex pheromone and a multi-scale approach to predict the distribution of a rare saproxylic beetle. PLoS One.

[CR42] Niehuis M (2001). Die Bockkäfer in Rheinland-Pfalz und im Saarland.

[CR43] Özdikmen H, Turgut S (2009). A short review on the genus *Plagionotus* Mulsant, 1842 (Coleoptera: Cerambycidae: Cerambycinae). Munis Entomol Zool.

[CR44] Rassati D, Marini L, Marchioro M, Rapuzzi P, Magnani G, Poloni R, Giovanni FD, Mayo P, Sweeney J (2018) Developing trapping protocols for wood-boring beetles associated with broadleaf trees. J Pest Sci. 10.1007/s10340-018-0984-y

[CR45] Ray AM, Millar JG, McElfresh JS, Swift IP, Barbour JD, Hanks LM (2009). Male-produced aggregation pheromone of the cerambycid beetle *Rosalia funebris*. J Chem Ecol.

[CR46] Ray AM, Arnold RA, Swift I, Schapker PA, McCann S, Marshall CJ, McElfresh JS, Millar JG (2014). (*R*)-Desmolactone is a sex pheromone or sex attractant for the endangered valley elderberry longhorn beetle *Desmocerus californicus dimorphus* and several congeners (Cerambycidae: Lepturinae). PLoS One.

[CR47] RStudio (2018) RStudio: Integrated development environment for R, version 1.1.453. https://www.rstudio.com/. Accessed 29 June 2018

[CR48] Schröder FC (1996) Identifizierung und Synthese neuer Alkaloide, Hydroxyketone und bicyclischer Acetale aus Insekten. PhD dissertation, University of Hamburg

[CR49] Švácha P, Lawrence JF, RAB L, Beutel RG (2014). Chapter 2.4 Cerambycidae Latreille, 1802. Handbook of zoology: Arthropoda: Insecta: Coleoptera, beetles. Vol. 3: Morphology and systematics (Phytophaga).

[CR50] Svensson GP, Sahlin U, Brage B, Larsson MC (2011). Should I stay or should I go? Modelling dispersal strategies in a threatened saproxylic beetle, *Osmoderma eremita*, based on pheromone capture and radio telemetry. Biodivers Conserv.

[CR51] Swedish Species Observation System (2015) List of records of *Plagionotus detritus* 2000–2015. https://www.artportalen.se/. Accessed 16 June 2015

[CR52] Torres-Vila LM, Zugasti C, De-Juan JM, Oliva MJ, Montero C, Mendiola FJ, Conejo Y, Sánchez Á, Fernández F, Ponce F, Espárrago G (2015). Mark-recapture of *Monochamus galloprovincialis* with semiochemical-baited traps: population density, attraction distance, flight behavior and mass trapping efficiency. Forestry.

[CR53] Torres-Vila LM, Mendiola-Diaz FJ, Sánchez-González Á (2017). Dispersal differences of a pest and a protected *Cerambyx* species (Coleptera: Cerambycidae) in oak open woodlands: a mark-recapture comparative study. Ecol Entomol.

[CR54] Xu T, Yasui H, Teale SA, Fujiwara-Tsujii N, Wickham JD, Fukaya M, Hansen L, Kiriyama S, Hao D, Nakano A, Zhang L, Watanabe T, Tokoro M, Millar JG (2017). Identification of a male-produced sex-aggregation pheromone for a highly invasive cerambycid beetle, *Aromia bungii*. Sci Rep.

[CR55] Zou Y, Rutledge CF, Nakamuta K, Maier CT, Hanks LM, Richards AB, Lacey ES, Millar JG (2016). Identification of a pheromone component and a critical synergist for the invasive beetle *Callidiellum rufipenne* (Coleoptera: Cerambycidae). Environ Entomol.

[CR56] Žunič Kosi A, Zou Y, Hoskovec M, Vrezec A, Stritih N, Millar JG (2017). Novel, male-produced aggregation pheromone of the cerambycid beetle *Rosalia alpina*, a priority species of European conservation concern. PLoS One.

